# Diagnostic value and risk correlation of serum heart-type fatty acid-binding protein (H-FABP) and osteoprotegerin (OPG) in chronic heart failure

**DOI:** 10.5937/jomb0-60310

**Published:** 2026-03-17

**Authors:** Zheng Weifeng, Liu Mingda, Xu Sanduo, Xu Wang

**Affiliations:** 1 Ningbo Fourth Hospital, Department of Cardiology, Ningbo, Zhejiang, China; 2 West China Hospital of Sichuan University, Department of Cardiology, Chengdu City, China; 3 Ganzhou People's Hospital (The Affiliated Ganzhou Hospital of Nanchang University), Department of Cardiovascular Medicine, Ganzhou City, China

**Keywords:** osteoprotegerin, NT-proBNP, chronic disease, cardiac function, sensitivity analysis, osteoprotegerin, NT-proBNP, hronična bolest, srčana funkcija, analiza senzitivnosti

## Abstract

**Background:**

To analyse the value of H-FABP and OPG in determining the severity of heart function in those suffering from long-term heart failure.

**Methods:**

A total of 196 patients with persistent heart failure who were admitted to our hospital between June 2023 and October 2024 were chosen. A comparison of the three groups of chronic heart failure patients' general clinical information and H-FABP and OPG levels was performed. The associations between OPG and H-FABP and various clinical forms of chronic heart failure, heart failure severity, and measurement markers related to echocardiography were examined. To assess the diagnostic utility of NT-proBNP, H-FABP, and OPG detection alone and in combination for HFrEF and HFpEF patients. All patients were monitored for three to six months following their discharge.

**Results:**

There was a positive correlation (r=0.61) between H-FABP and the NYHA classification, LA (r=0.46), LV (r=0.51), HS-TnT (r=0.31), NT-proBNP (r=0.58), SUA (r=0.38), etc., and negatively correlated with the LVEF (r=-0.76), NT-proBNP (r=0.49), etc., and negatively correlated with the LVEF (r=-0.60) (P&lt;0.05). Binary logistic regression analysis of NT-proBNP, H-FABP, and OPG levels and endpoint events revealed that NT-proBNP had a high value in predicting endpoint events. The readmission rate and mortality rate of patients with chronic heart failure increased with increasing NT-proBNP concentration. Serum H-FABP and OPG all have high diagnostic value for HFrEF. Compared with the traditional biomarker NT-proBNP, H-FABP had greater sensitivity (94.9%) and specificity (83.1%) in the diagnosis of HFrEF, whereas OPG had greater sensitivity (92.3%) and lower specificity (57.7%). H-FABP and OPG can significantly improve the sensitivity (86.44%) and specificity (89.74%) in the diagnosis of patients with HFrEF. Serum NT-proBNP, H-FABP and OPG all have high diagnostic value for HFpEF. Compared with the traditional biomarker NT-proBNP, H-FABP has greater sensitivity (91.7%) and specificity (82.0%) in the diagnosis of HFpEF, whereas OPG has greater specificity (82.0%) and lower sensitivity (58.3%).

**Conclusions:**

The combined detection of H-FABP, OPG, and NT-proBNP can be used as an essential strategy for the early detection of decreased cardiac function in heart failure patients.

## Abstract (sr)

**Uvod:**

Cilj je bio da se analizira značaj H-FABP i OPG u određivanju težine oštećenja srčane funkcije kod pacijenata sa hroničnom srčanom insuficijencijom.

**Metode:**

U istraživanje je uključeno 196 pacijenata sa perzistentnom srčanom insuficijencijom, hospitalizovanih u našoj ustanovi u periodu od juna 2023. do oktobra 2024. godine. Analizirani su opšti klinički podaci i nivoi H-FABP i OPG u tri grupe pacijenata sa hroničnom srčanom insuficijencijom. Ispitivane su povezanosti H-FABP i OPG sa različitim kliničkim oblicima hronične srčane insuficijencije, stepenom srčanog popuštanja i parametrima dobijenim ehokardiografijom. Takođe je procenjivana dijagnostička vrednost pojedinačnog i kombinovanog određivanja NT-proBNP H-FABP i OPG kod pacijenata sa HFrEF i HFpEF Svi pacijenti su praćeni tokom perioda od tri do šest meseci nakon otpusta.

**Rezultati:**

Utvrđena je pozitivna korelacija između H-FABP i NYHA klasifikacije (r=0,61), LA (r=0,46), LV (r=0,51), HS-TnT (r=0,31), NT-proBNP H-FABP i OPG nivoa i krajnjih događaja pokazala je da NT-proBNP ima visoku prediktivnu vrednost za krajnje događaje. Stopa rehospitalizacije i mortaliteta kod pacijenata sa hroničnom srčanom insuficijencijom rasla je sa porastom koncentracije NT-proBNP Serum H-FABP i OPG pokazali su visoku dijagnostičku vrednost za HFrEF. U poređenju sa tradicionalnim biomarkerom NT-proBNP H- FABP je imao veću senzitivnost (94,9%) i specifičnost (83,1%) u dijagnostici HFrEF dok je OPG imao veću senzitivnost (92,3%) i nižu specifičnost (57,7%). Kombinacija H-FABP i OPG značajno poboljšava senzitivnost (86,44%) i specifičnost (89,74%) u dijagnostici pacijenata sa HFrEF Serum NT-proBNP H-FABP i OPG takođe imaju visoku dijagnostičku vrednost za HFpEF U poređenju sa NT- proBNP H-FABP je imao veću senzitivnost (91,7%) i specifičnost (82,0%) u dijagnostici HFpEF dok je OPG imao veću specifičnost (82,0%) i nižu senzitivnost (58,3%).

**Zaključak:**

Kombinovano određivanje H-FABP OPG i NT-proBNP se može koristiti kao značajna strategija za rano otkrivanje smanjene srčane funkcije kod pacijenata sa srčanom insuficijencijom.

## Introduction

The term heart failure describes a collection of diseases characterised by inadequate perfusion of tissues and organs, congestion in the pulmonary and/or systemic circulation, and reduced ventricular filling and ejection functions as a result of aberrant cardiac structure and function [1][2][3]. The main clinical manifestations are limited physical activity, fluid retention and breathing difficulties. Heart failure, the greatest challenge in cardiology-related diseases in the 21st century, is positively correlated with age [4]. Additionally, the annual death rate for people with chronic heart failure increases by approximately 2.8% as they grow older and other risk factors increase [5]. Despite notable advancements in the diagnosis and treatment of heart failure in recent years, patients with chronic heart failure continue to have high rates of death and readmission. Heart failure patients have a 25% chance of being readmitted to the hospital within a month and a 50% chance of dying within five years of receiving a chronic heart failure diagnosis [6][7][8]. Chronic heart failure has emerged as a significant public health issue as the population ages [9].

Heart failure is a severe manifestation and advanced stage of all cardiovascular diseases. Heart failure patients’ frequent hospital stays are significant indicators of older patients’ repeated hospital stays [10]. As more people are admitted to hospitals for heart failure, the patient mortality rate increases [11]. Heart failure inpatients and those in the stable stage of the disease had one-year readmission rates of 44% and 32%, respectively. The leading cause of death for both hospitalised and non-hospitalised patients is heart disease, which mainly manifests as sudden cardiac death and malignant heart failure [12]. Therefore, early detection and treatment of heart failure, as well as classification, especially in elderly patients, can reduce the readmission rate and long-term mortality rate of heart failure.

Studies [13][14][15][16] have shown that H-FABP in peripheral blood can increase within 15 minutes after myocardial infarction and return to normal levels within approximately 20 hours. Osteoprotegerin (OPG) is a fundamental secretory glycoprotein composed of 401 amino acids and exists mainly in the form of a 60 kDa monomer or a 120 kDa dimer in the peripheral circulation [17]. Patients with chronic heart failure for three to six months were followed to investigate the connections between H-FABP and OPG, the degree of cardiac function, and other clinical categories of heart failure, with events such as cardiogenic death, readmission due to acute heart failure, recurrent heart failure, and malignant arrhythmia as endpoint events. To analyse the influence of H-FABP and OPG levels on clinical prognosis.

## Materials and methods

### Research subjects and groups

We selected 110 patients with persistent heart failure who were admitted to our facility between June 2023 and October 2024. A total of 196 patients, 126 men and 70 women, were enrolled after screening according to the inclusion and exclusion criteria.

Inclusion criteria:

Symptoms and signs of chronic heart failure, such as dyspnea, orthostatic breathing, reduced activity tolerance, and ankle oedema; NYHA grade I-IV; (II) Echocardiography suggesting chronic heart failure and meeting one of the following conditions.

Exclusion criteria:

Under 18 years of age; (II) acute or chronic infection; (III) malignant tumour; (IV) renal insufficiency; (V) hyperthyroidism and other special types of diseases.

Three categories were created based on the NYHA cardiac function classification standard: NYHA grade II, NYHA grade III, and NYHA Grade IV. The levels of H-FABP, OPG, NT-proBNP and other biochemical indicators and cardiography-related indicators measured by echocardiography were compared among the groups.

### Collection of basic data and biochemical indicators

Using our hospital’s electronic information system, the baseline characteristic data of all patients who met the standards, including name, sex, age, height, weight, body surface area, diagnosis, and comorbidities, were obtained.

The complete biochemical indicators measured within 24 hours of admission included HS-TnT, NT-proBNP, the percentage of neutrophils, and albumin. All patients had their venous blood drawn for testing within 24 hours after admission. It was essential to inform the patients to fast.

### Determination of H-FABP and OPG data

All the selected patients with heart failure had 2–3 mL of venous blood drawn into the procoagulation tube after fasting for 6–8 hours in the early morning of the second day after admission. The supernatant was centrifuged at 4000 rpm for 5 minutes, drawn with a pipette and stored at -80°C. H-FABP and OPG levels were determined via enzyme-linked immunosorbent assay.

### Statistical methods analysis

The rank sum test was used for grade data. Since there is a single frequency of 0, Fisher’s exact test is adopted. Correlation analysis can be conducted via Pearson’s or Spearman’s test. Multivariate regression analysis and binary logistic regression analysis were applied to classify cardiac function and analyse the influencing factors of different types of heart failure. The rehospitalisation rate and mortality rate were analysed via the Cox regression method.

## Results

### Comparison of the general data of patients in different groups according to heart failure type

The patients in the HFrEF group were younger. Compared with those of patients in the HFmrEF and HFpEF groups, the left atrial and left ventricular inner diameters and LVMI values of HFrEF patients were greater, whereas the left atrial diameter of HFmrEF patients was greater than that of HFpEF patients. The OPG concentration in the peripheral blood of HFmrEF and HFpEF patients was lower (Table 1).

**Table 1:** Baseline data of the CHF groups.

	HFrEF, N=78	HFmrEF, N=22	HFpEF, N=96	P value
Age (year)	53.41±13.58	56.18±21.54	63.88±11.28	P<0.05
Gender (Male/Female)	54/24	16/6	54/40	P=0.542
Complications (%)				P=0.128
T2DM	16 (20.5%)	2 (9.1%)	6 (6.3%)	-
Hypertension	28 (35.9%)	10 (45.5%)	46 (47.9%)	-
T2DM & Hypertension	2 (2.6%)	2 (9.1%)	12 (12.5%)	-
NYHA Classification (%)				P<0.05
level	8 (10.3%)	4 (18.2%)	56 (58.3)	P>0.05
level	50 (64.1%)	16 (72.7%)	40 (41.7)	P<0.05
IV level	20 (25.6%)	2 (9.1%)	0	P>0.05
LA (mmol/L)	4.79±0.62	4.44±0.62	4.12±0.66	P<0.05
LV (mL)	6.50±1.04	5.47±0.64	5.00±0.18	P<0.05
LVMI (g/m^2^)	147.97±49.22	121.87±29.50	104.12±25.00	P<0.05
NPAR (10^9^/L)	1.62±0.39	1.64±0.36	1.62±0.37	P=0.908
GHbA1 (%)	6.82±1.31	6.38±1.07	6.67±1.12	P=0.288
D-Dimer (ng/mL)	1.09±1.33	2.30±4.07	0.78±1.23	P=0.112
CHOL (mmol/L)	4.47±1.50	4.53±1.19	3.83±1.38	P<0.05
LDL-C (mmol/L)	2.81±1.06	2.63±0.74	2.25±0.93	P<0.05
HDL-C (mmol/L)	1.15±0.31	1.38±0.58	1.15±0.35	P=0.067
LP(a) (nmol/L)	0.28±0.34	0.13±0.10	0.27±0.26	P=0.247
SUA (μmol/L)	497.87±140.64	502.00±162.23	409.02±133.17	P<0.05
tHCY (μmol/L)	18.17±13.83	18.68±5.44	19.54±25.03	P=0.232
CREA (μmol/L)	82.08±23.82	88.25±29.22	75.49±18.45	P=0.214
CysC (mg/L)	1.19±0.37	1.41±0.42	1.18±0.33	P=0.193
HS-TnT (ng/L)	141.14±462.40	67.89±148.78	46.94±94.41	P=0.079
NT-proBNP (pg/mL)	3458.56±3796.35	2434.07±2333.18	1200.51±2118.92	P<0.05
H-FABP (ng/mL)	11.49±1.95	8.48±3.56	5.80±2.70	P<0.05
OPG (ng/mL)	1.70±0.55	1.19±0.56	1.03±0.42	P<0.05

### Correlation analysis of H-FABP and OPG with biochemical indicators, cardiac ultrasound and NYHA classification

The levels of OPG and H-FABP in the peripheral blood of individuals with chronic heart failure were correlated with clinical biochemical markers and indicators linked to echocardiography (r<0.3 was considered a weak correlation and was not considered). Pearson correlation analysis was used if both variables followed a normal distribution. For bivariate nonnormally distributed or graded data, Spearman correlation analysis was applied (Table 2).

**Table 2:** Correlation analysis of H-FABP and OPG with age, biomarkers, echocardiography and NYHA grade.

	H-FABP		OPG	
	r	P	r	P
Age	-0.24	=0.020	-0.04	=0.587
NYHA classification	0.61	<0.001	0.60	<0.001
LA	0.51	<0.001	0.36	<0.001
LV	0.46	<0.001	0.32	<0.001
IVS	-0.23	=0.024	-0.25	=0.012
LVPW	-0.13	=0.065	-0.22	=0.030
LVEF	-0.76	<0.001	-0.60	<0.001
LVMI	0.30	=0.003	0.22	=0.026
HS-TnT	0.31	=0.002	0.12	=0.086
NT-proBNP	0.58	<0.001	0.49	<0.001
SUA	0.38	<0.001	0.15	=0.027
CREA	0.26	=0.011	0.21	=0.038

Peripheral blood H-FABP was positively correlated with NT-proBNP (r=0.58), SUA (r=0.38), HS-TnT (r=0.314), LA (r=0.51), LV (r=0.46), and NYHA cardiac function classification (r=0.48) (P<0.05). The correlation between this parameter and the LVEF was negative (r=-0.761, P<0.01). On the other hand, OPG and the NYHA cardiac function categorisation showed a favourable correlation (r=0.48), LA (r=0.51), LV (r=0.32), NT-proBNP (r=0.49), etc. (P<0.05) (Table 3).

**Table 3:** Correlation analysis of NT-proBNP, H-FABP and OPG on NYHA grade.

NYHA Classification	NT-proBNP (pg/mL)	H-FABP (ng/mL)	OPG (ng/mL)
NYHA Class II	685.58±228.37	5.63±0.58	0.88±0.65
NYHA Class III	2727.89±378.52	9.37±0.40	1.49±0.75
NYHA Class IV	4672.26±1678.96	12.02±0.61	1.79±0.13

### The influence of H-FABP and 0PG on NYHA cardiac function classification

Excluding other possible influencing factors, H-FABP in the serum of NYHA Grade III patients was significantly greater than that in the serum of NYHA Grade II patients. Moreover, for every lng/mL increase in H-FABP, the risk of NYHA grade III disease in patients increased by 2.13 times (95% CI (1.099, 4.112)). Excluding other possible influencing factors, compared with NYHA Grade II patients, NYHA Grade IV patients had noticeably higher serum H-FABP levels. Moreover, for every 1 ng/mL increase in H-FABP, the risk of NYHA grade IV disease in patients increased 2.95-fold (95% CI (1.429, 6.072), P<0.05) (Table 4).

**Table 4:** Multivariate regression analysis of H-FA BP and OPG with different NYHA cardiac function grades.

NYHA classification	Parameter	B	SE	Exp(B)	P value	95%CI
NYHA Class II	H-FABP	0.636	0.327	1.888	0.052	(0.995, 3.584)
	OPG	-0.043	1.588	0.958	0.978	(0.043, 21.512)
	NT-proBNP	0.000	0.001	1.000	0.736	(0.998, 1.003)
NYHA Class III	H-FABP	0.754	0.337	2.126	0.025	(1.099, 4.112)
	OPG	2.252	1.656	9.506	0.174	(0.370, 243.935)
	NT-proBNP	0.001	0.001	1.001	0.564	(0.998, 1.004)
Nyha Class IV	H-FABP	1.080	0.369	2.946	0.003	(1.429, 6.072)
	OPG	2.863	1.775	17.510	0.107	(0.540, 567.584)
	NT-proBNP	0.001	0.001	1.001	0.530	(0.998, 1.004)

### The influence of H-FABP and OPG on different types of heart failure

In patients with HFrEF, the peripheral blood H-FABP concentration was greater than that in patients with HFpEF. Additionally, patients were 2.37 times more likely to present with HFrEF for every Ing/mL increase in H-FABP (95% CI (1.596, 3.529), P<0.001). Patients with heart failure had higher peripheral blood levels of H-FABP. Additionally, patients’ risk of HFmrEF increased 1.47 times for every g/mL increase in H-FABP (95% CI (1.055, 2.040), P<0.05). However, no discernible difference was found in the distribution of OPG levels between HFpEF patients and HFmrEF patients (Table 5).

**Table 5:** Analysis of H-FABP, OPG, and various HF types using multiple regression.

Chronic heart failure	Parameter	B	SE	Exp(B)	P value	95%CI
HFrEF	H-FABP	0.864	0.202	2.373	<0.001	(1.596, 3.529)
	OPG	1.081	0.803	2.949	0.178	(0.611, 14.228)
	NT-proBNP	0.000	0.000	1.000	0.736	(1.000, 1.000)
HFmrEF	H-FABP	0.383	0.168	1.467	0.023	(1.055, 2.040)
	OPG	-0.456	0.940	0.634	0.628	(1.100, 4.005)
	NT-proBNP	0.000	0.000	1.000	0.686	(1.000, 1.000)

### Differences in the distributions of NT-proBNP, HFABP and OPG in heart failure patients with different etiologies

Chronic heart failure patients were split into a group based on myocardial lesions in this study (N=86), the valvular heart failure group (N=16), the ischemic cardiomyopathy group (N=62), and other cause groups (including severe myocarditis, hypertensive heart disease, etc.) (N=16) based on the cause of heart failure. Baseline data, ultrasound indicators, OPG and H-FABP in each group revealed that no notable variations were observed in patients with chronic heart failure in terms of sex, IVS, LVPW, comorbidities, HS-TnT, NT-proBNP, NPAR, GHbA1, CHOL, LDL-C, HDL-C, LP(a), tHCY, SUA, CREA, CysC, D-dimer or OPG (Table 6).

**Table 6:** The distribution of H-FABP, age, LVEF, LV and LA in different causes of heart failure.

	Myocardial<br>lesions	Valvular heart<br>failure	Ischemic<br>cardiomyopathy	Other<br>reasons	P value
Age (year)	54.4±15.28	64.38±13.88	60.74±12.71	64.38±12.57	0.039
LA (mmol/L)	4.62±0.68	2.88±0.64	2.39±0.80	2.81±0.75	0.000
LV (mL)	6.12±1.21	5.09±0.82	5.37±0.69	5.20±1.07	0.001
LVEF (%)	37.72±12.05	53.13±6.94	51.42±11.19	45.81±11.59	0.000
LVMI (g/m^2^)	252.40±100.29	192.34±56.64	201.39±51.36	204.73±83.48	0.026
NT-proBNP<br>(pg/mL)	2941.53±3696.02	2208.88±2254.39	1614.51±2780.21	1567.25±1664.78	0.236
OPG (ng/mL)	1.25±0.43	1.46±0.38	1.35±0.57	1.53±0.59	0.263
H-FABP (ng/mL)	7.41±3.22	11.82±3.99	6.81±3.75	8.45±2.87	0.016

### Analysis of the effects of OPG, H-FABP, and NT-proBNP on endpoint events via binary regression

Among the 196 patients included and followed up for 1 to 6 months (with an average of 17 weeks), 6 patients died, and 28 patients were rehospitalised for treatment. Binary logistic regression analysis was conducted by defining death and rehospitalisation as the composite endpoint events as the dependent variables and NT-proBNP, OPG, H-FABP, age, and comorbidities as independent variables. Factors such as OPG, H-FABP, age, and comorbidities could not predict patient prognosis (P>0.05). Patients with chronic heart failure and their prognosis were significantly better predicted by the traditional heart failure indicator NT-proBNP (P<0.05) (Table 7).

**Table 7:** Binary logistic regression analysis of NT-proBNP, OPG, H-FABP, age.

Variable	B	Standard error	P value	Risk ratio	95% CI
H-FABP	-0.02	0.08	0.809	0.98	(0.83, 1,16)
OPG	0.47	0.57	0.417	1.59	(0.52, 4.91)
NT-proBNP	0.00	0.00	0.037	1.00	(1.00, 1.00)
Complications	-0.47	0.57	0.407	0.62	(0.20, 1.90)
Age	0.05	0.59	0.936	1.05	(0.33, 3.35)

Compared with patients with chronic heart failure whose peripheral blood NT-proBNP concentration was less than 1078 pg/mL, those whose concentration was greater had a higher readout rate and a higher probability of dying. Consequently, the measurement of NT-proBNP levels in the peripheral blood of CHF patients who have been admitted serves as an independent predictor of patients’ outcome events (Table 8).

**Table 8:** Hospitalisation rate and mortality of CHF patients with different concentrations of NT-proBNP.

Grouping	n	Deceased<br>patient	Mortality rate<br>(%)	Rehospitalised<br>patients	Readmission rate<br>(%)
NT-proBNP 1078 pg/mL	88	4	4.5	22	25.0
NT-proBNP<1078 pg/mL	108	2	1.8	6	5.5
** Total **	** 196 **	** 6 **	** 3.0 **	** 28 **	** 14.3 **

## Discussion

The distributions of NT-proBNP, H-FABP and OPG also vary among chronic heart failure patients with different NYHA grades [18]. These novel biomarkers suggest that H-FABP and OPG are crucial for determining the degree of cardiac function in individuals suffering from chronic heart failure. The diagnosis of HFrEF and HFpEF can be made with much greater sensitivity and specificity when the conventional biomarkers NT-proBNP, H-FABP, and OPG are detected together [19]. The risk of NYHA grade III was 2.13 times greater for patients with elevated H-FABP levels than for those with NYHA grade II, and the risk of NYHA grade IV was 2.95 times greater for those with elevated H-FABP levels (95% CI (1.429, 6.072), P<0.05). Thus, H-FABP is directly linked to the reduced cardiac function of individuals with chronic heart failure and is an independent risk factor for more severe cardiac insufficiency in these patients [20][21][22]. In addition, multivariate regression analysis of different types of heart failure revealed that, after other possible influencing factors were excluded [23]. Moreover, patients with elevated H-FABP levels had a 2.37-fold increased risk of HFrEF (95% CI (1.596, 3.529), P<0.05). However, NT-proBNP and OPG showed no significant abnormalities in patients with different types of heart failure [24].

Its primary mode of action is connected to OPG activation of the RAAS system, the deposition of many collagen fibres and the extracellular matrix in cardiomyocytes and interstitial tissue, and the activation of IL-17 to participate in the regulation of myocardial fibrosis, which plays a complex role in ventricular remodelling [25][26][27]. At present, the critical indicators for evaluating ventricular remodelling in actual clinical work are the left atrium, left ventricle and left ventricular mass indices via echocardiography, etc. [28]. The LV and LVMI values of HFrEF patients were both greater than those of HFmrEF and HFpEF patients [29]. Moreover, the changes in ventricular structures such as LA, LV and LVMI in HFrEF patients are more significant, which leads to uncoordinated cardiac systolic function and poorer cardiac function in HFrEF patients, who are unable to meet the demands of the body’s tissues and organs [30][31][32].

In patients with chronic heart failure, new cardiac biomarkers, such as FABP and OPG, are essential for assessing the level of left ventricular systolic dysfunction and cardiac function.

## Dodatak

### Conflict of interest statement

All the authors declare that they have no conflict of interest in this work.
